# Tetrahydrobiopterin induces proteasome inhibitor resistance and tumor progression in multiple myeloma

**DOI:** 10.1007/s12032-021-01632-5

**Published:** 2022-02-12

**Authors:** Hua Zhang, Jintong Chen, Mingyue Zhang, Munan Zhao, Luyao Zhang, Bin Liu, Siqing Wang

**Affiliations:** 1 Department of Gastrointestinal Surgery, The First Hospital of Jilin University, Changchun, 130021 China; 2 Department of Cancer Immunology, The First Hospital of Jilin University, 519 Dongminzhu St, Changchun, 130061 Jilin China; 3 Department of Gynecological Oncology, The First Hospital of Jilin University, Changchun, 130021 China; 4 Department of Cancer Center, The First Hospital of Jilin University, Changchun, 130021 China; 5 Department of Hand and Foot Surgery, The First Hospital of Jilin University, 71 Xinmin St, Changchun, 130021 Jilin China

**Keywords:** Multiple myeloma, Tetrahydrobiopterin, USP7, NF-κB, p53

## Abstract

Multiple myeloma (MM) still remains an incurable disease due to widespread drug resistance and high frequency of relapse. In this study, we found that tetrahydrobiopterin (BH4) promotes MM cell proliferation and tumor growth in vivo. BH4 also increases MM bortezomib (Bor) resistance in vitro and in vivo. We show that BH4 increases the expressions of USP7 and USP46 in MM cells, which are responsible for MM Bor resistance primed by BH4. BH4 promotes the degradation of P53 and the activation of NF-κB signaling through the up-regulation of USP7 and USP46. Furthermore, the inhibition of USPs increases the therapeutic effects of Bor in MM tumor bearing mice. Our results demonstrate the important role of BH4 in MM Bor resistance and tumor progression in vivo. These findings could potentially have clinical implications.

**Supplementary Information:**

The online version contains supplementary material available at 10.1007/s12032-021-01632-5.

## Background

Multiple myeloma (MM) is a plasma cell malignancy mainly residing in the bone marrow [1]. Recently, new chemotherapeutic drugs and regimens have substantially improved the prognosis of patients with MM, with an increase in median survival from 3–5 years to 8–10 years [2, 3]. However, MM still remains largely incurable. Most patients eventually relapse due to chemotherapeutic drug resistance [4]. Therefore, the investigation of the mechanisms underlying MM drug resistance may have important clinical significance.

Proteasome inhibitors, such as bortezomib (Bor) and carfilzomib, are highly effective and widely used for the treatment of MM [5–7]. The ubiquitination and degradation of p53 and IκB plays an important role in MM cell survival and tumor progression [8, 9]. Proteasome inhibitors promote the apoptosis of MM cells through inhibiting proteasome-mediated degradation of the ubiquitinated p53 and IκB [7, 10]. Although proteasome inhibitors have achieved clinical success, most patients eventually develop resistance to the therapy [11, 12]. And the underlying mechanisms need to be further investigated.

Tetrahydrobiopterin (BH4) is a co-factor of nitric oxide synthase (NOS), tyrosine hydroxylase, and tryptophan hydroxylase, which are involved in the biosynthesis of nitric oxide and monoamine neurotransmitters and pain sensitivity [13]. BH4 promotes tumor growth and modulates ubiquitination and proteosome activity through NOS-mediated S-nitrosation of target proteins [14, 15]. Loss of BH4 triggers the increase of ubiquitinated proteins and subsequent degradation in cells [16, 17]. However, the role of BH4 in MM drug resistance and progression remains unknown.

In this study, we found that BH4 promotes MM cell proliferation and tumor growth in vivo. And, BH4 treatment inhibits Bor-induced antitumor effects. We show that BH4 upregulates the expressions of USP7 and USP46 in MM cells, which are essential for BH4-induced MM Bor resistance. BH4 increases p53 degradation and activates NF-kB signaling pathways in a USP-dependent manner. Furthermore, the inhibition of USPs increases the therapeutic effects of Bor in MM tumor bearing mice. Our results demonstrate the important role of BH4 in MM Bor resistance and tumor progression and may have important clinical implications.

## Methods

### Mice and cell lines

Balb/c mice were purchased from the Jackson Laboratory and bred in pathogen-free facilities at the First Hospital Animal Center of Jilin University. Mice were used in experiments at 6–8 weeks of age. All animal experimental procedures were reviewed and approved by the Animal Ethical Committee of First Hospital of Jilin University.

MPC-11, MOPC-315, ARP-1, and CAG MM cell lines were purchased from ATCC (Rockville, MD) and were cultured in RPMI 1640 medium supplemented with 10% heat-inactivated fetal bovine serum (FBS, ExCell), 100 U/mL penicillin (Invitrogen), and 100 mg/mL streptomycin (Invitrogen). Cells were grown in standard (37 °C, 5% CO_2_) culture incubators.

### Reagents and antibodies

BH4 was purchased from Sigma. BH4 was dissolved in RPMI 1640 medium or PBS to a stock concentration of 20 mM. Bor was purchased from Selleckchem. P22077 (USP inhibitor, USPi) was purchased from MedChemExpress. Bor and P22077 were dissolved in dimethylsulfoxide (DMSO) to stock concentrations of 80 mM and 150 mM. For cell assays, BH4 was used at a concentration of 40 μM, Bor at 15 nM, and P22077 at 10 μM. For in vivo use, BH4, Bor, and P22077 were injected intraperitoneally (i.p.) at doses of 60 μg/mouse, 20 μg/mouse, and 200 μg/mouse.

### In vivo functional tests

In MM cell proliferation tests, MPC-11 and MOPC-315 MM cells (1 × 10^6^ per mouse) were labeled with CFSE and injected into Balb/c mice through the tail veins. Mice were injected i.p. with BH4 (60 μg/mouse) on day 0, day 2, and day 4 after tumor challenge. Mice treated with PBS served as controls. On day 5 after tumor challenge, cells were isolated from lung tissues and analyzed by flow cytometry.

In MM Bor resistance and tumor progression tests, 1 × 10^6^ MPC-11 or MOPC-315 cells were injected subcutaneously (s.c.) into Balb/c mice. From day 0 or 6 after tumor challenge, mice were randomly divided into groups and given treatments every 2 days. Mice injected with PBS served as controls. Tumor development was monitored over time. Tumor volume was calculated by the formula: 3.14 × (mean diameter)^3^/6. When the tumor diameter reached to the range between 1.5 and 2 cm, mice were killed by cervical dislocation under ketamine anesthesia.

### Flow cytometry

Flow cytometry were performed as described previously [18, 19]. FITC-Annexin V and PI were purchased from BD Biosciences and performed to test cell viability. CFSE (carboxyl fluorescein diacetate, succinimidyl ester) was purchased from Invitrogen and used to measuring cell proliferation. Cells were analyzed by a BD LSRFortessa™ cytometer.

### Quantitative polymerase chain reaction (qPCR)

Total RNA was extracted from cells using an EasyPure RNA Kit (TransGen Biotech), and cDNA was synthesized with an All-in-One First-Strand cDNA Synthesis SuperMix (TransGen). The mRNA levels of *Usp7* and *Usp46* by MPC-11 and MOPC-315 cells were analyzed. Expression was normalized to the expression of the housekeeping gene *Gapdh*. Primer sets used for these analyses are listed as follows: *Gapdh*, 5′-AGC TTG TCA TCA ACG GGA AG-3′ (forward) and 5′-TTT GAT GTT AGT GGG GTC TCG-3′ (reverse); *Usp7*, 5′-AAG TCT CAA GGT TAT AGG GAC GG-3′ (forward) and 5′-CCA TGC TTG TCT GGG TAT AGT GT-3′ (reverse); *Usp46*, 5′-ATG ACT GTC CGA AAC ATC GCC-3′ (forward) and 5′-TTG ACC AAT CC GAA GTA GTG TTC-3′ (reverse).

### RNA interference

RNA interference was performed as previously described [18]. Day 0 MPC-11 cells were transfected with 100 nM siRNA with transfection reagent RNAFit (HANBIO) according to the manufacturer’s protocol. Silencing was confirmed at the mRNA levels by qPCR on day 1. Transfected MPC-11 cells were treated with BH4 + Bor for 24 h. On day 2, MPC-11 cells were collected and analyzed by flow cytometry staining. siRNAs used are listed as follows: *Control* 5′-UUC UCC GAA CGU GUC ACG UTT-3′ (sense) and 5′-ACG UGA CAC GUU CGG AGA ATT-3′ (anti-sense); *Usp7*, 5′-GGU GGA ACG AUU GCA AGA ATT-3′ (sense) and 5′-UUC UUG CAA UCG UUC CAC CTT-3′ (anti-sense); *Usp46*, 5′-GCA UUA CAU CAC CAU CGU ATT-3′ (sense) and 5′-UAC GAU GGU GAU GUA AUG CTT-3′ (anti-sense).

### Western-blot analyses

Whole-cell lysates were harvested by RIPA Buffer (cat #: 9806, CST) containing Protease/Phosphatase Inhibitor Cocktail (100X) (cat #: 5872, CST). The protein lysates were resolved on 12% polyacrylamide sodium dodecyl sulfate (SDS) gels. Anti-mouse p53, caspase3, β-actin, p-IκBα, IκBα, NF-κB1 p105/50, NF-κB p65, and Histone H3 antibodies were purchased from Cell Signaling Technology (CST). Nuclear protein extraction kit (cat #: DE201-01) was purchased from TransGen. Images have been cropped for presentation.

### RNA sequencing (RNA-Seq)

MPC-11 and MOPC-315 cells were cultured with or without (PBS) the addition of BH4 for 24 h and cells were collected for RNA extraction. Total RNA was extracted with the Trizol (Thermo Fisher) and RNA-Seq was done by Sangon Biotech (Shanghai, China).

### Statistical analysis

The Student *t* test (2 groups) and ANOVA (≥ 3 groups) were used to compare various experimental groups. A *P* value of less than 0.05 was considered significant.

## Results

### BH4 promotes MM progression in vivo

**Fig. 1:**
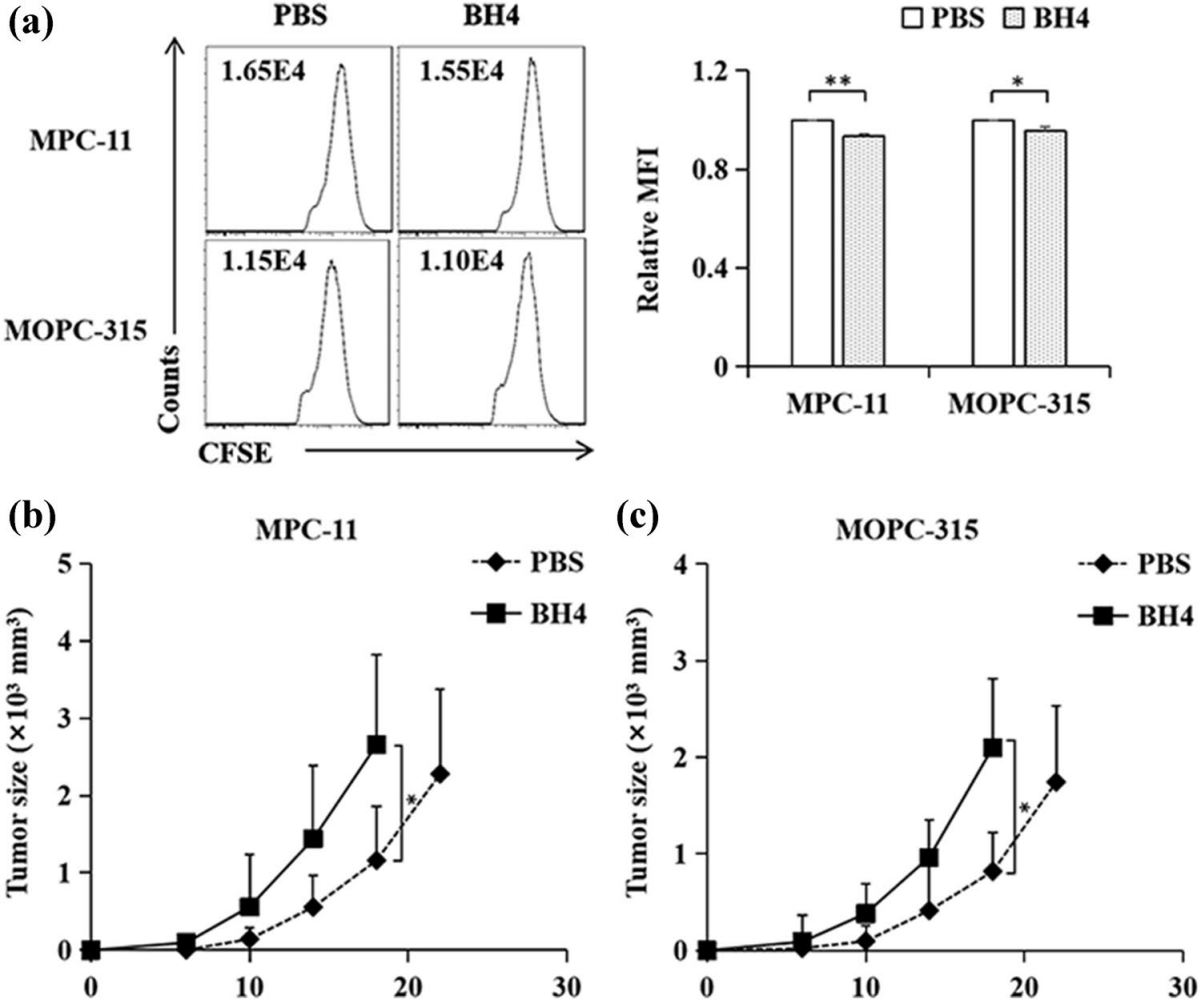
BH4 promotes MM progression in vivo. **a** MPC-11 and MOPC-315 MM cells (1 × 10^6^ per mouse) were labeled with CFSE and injected into Balb/c mice through the tail veins. Mice were injected intraperitoneally (i.p.) with BH4 (60 μg/mouse) on day 0, day 2, and day 4 after tumor challenge. Mice treated with PBS served as controls. On day 5, cells were isolated from lung tissues and analyzed by flow cytometry. Numbers in the histograms represent the fluorescence intensity (FI). Right, summarized results of three independent experiments obtained as the left. *MFI* mean fluorescence intensity. Data are presented as mean ± SD of three independent experiments. **b**, **c** Balb/c mice were injected subcutaneously (s.c.) with MPC-11 (**b**) or MOPC-315 (**c**) cells (1 × 10^6^ per mouse). From day 0 after tumor challenge, mice were injected i.p. with BH4 every 2 days. Mice treated with PBS served as controls. Shown are the tumor growth curves. The experiments were performed twice with a total of 10 mice per group (*n* = 10). Data are presented as mean ± SD. *NS* not significant. *P < 0.05; **P < 0.01

We first examined the role of BH4 treatment in MM cell proliferation in vivo, CFSE-labeled MPC-11 cells were injected intravenously (i.v.) into Balb/c mice. Mice were treated with BH4 on day 0, 2, and 4 after tumor injection. BH4 treatment increased MM cell proliferation as compared with PBS control (Fig. 1a). Similarly, BH4 treatment induced more potent cell proliferation responses than PBS control in MOPC-315 MM mouse model (Fig. 1a). These data indicated that BH4 stimulates MM cell proliferation in vivo.

We next examined the effects of BH4 treatment on MM tumor growth. MPC-11 cells were injected s.c. into Balb/c mice and mice were treated with BH4 every 2 days after tumor challenge. BH4 promoted MM tumor growth as compared with PBS control (Fig. 1b). In addition, BH4 treatment also stimulated MOPC-315 MM tumor growth in Balb/c mouse model (Fig. 1c). Together, these results demonstrates that BH4 promotes MM progression in vivo.

### BH4 increases Bortezomib resistance in MM

**Fig. 2:**
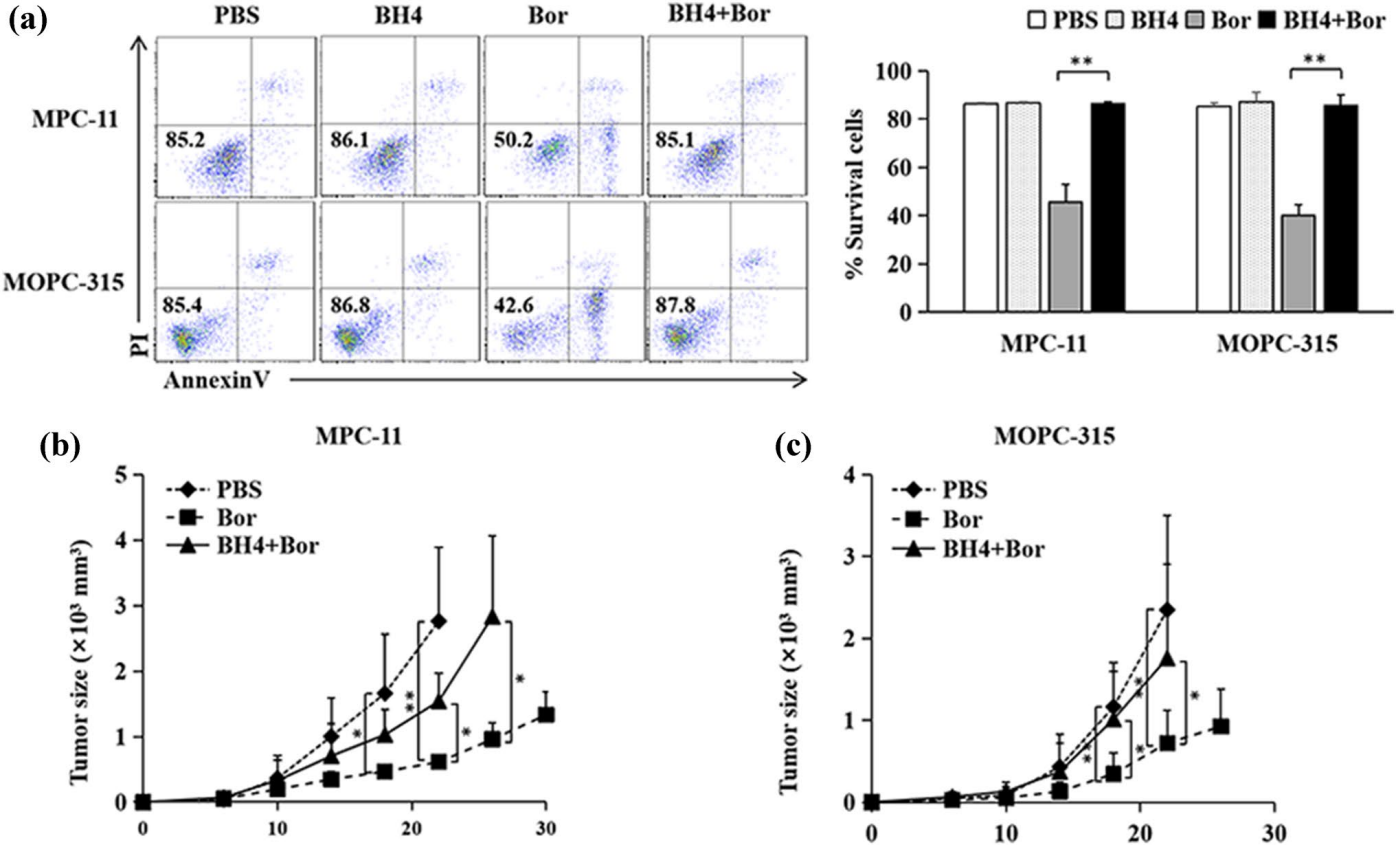
BH4 increases Bortezomib resistance in MM. **a** MPC-11 and MOPC-315 cells were cultured in the presence of BH4, Bortezomib (Bor), or their combinations (BH4 + Bor) for 24 h. Cells treated with PBS served as controls. Cell apoptosis was analyzed by flow cytometry. Numbers in the dot plots represent viability of MM cells. Showing are representative data of three independent experiments. Right, summarized results of three independent experiments obtained as left. Data are presented as mean ± SD. **b**, **c** Balb/c mice were injected s.c. with MPC-11 (**b**) or MOPC-315 (**c**) cells (1 × 10^6^ per mouse). From day 6 after tumor challenge, mice were injected i.p. with Bor or BH4 + Bor every 2 days. Mice received PBS served as controls. Shown are the tumor growth curves. The experiments were performed twice with a total of 10 mice per group (*n* = 10). Data are presented as mean ± SD. *P < 0.05; **P < 0.01

We next examined the effects of BH4 on Bor-induced MM cell apoptosis. As shown in Fig. 2a, while the addition of Bor efficiently induced MPC-11 MM cell apoptosis in the cell culture as compared with PBS control, the addition of BH4 significantly inhibited Bor-induced MPC-11 MM cell apoptosis. Furthermore, the addition of BH4 inhibited Bor-induced cell apoptosis in MOPC-315 MM cell model (Fig. 2a). We also examined the effects of BH4 on Bor-induced cell apoptosis in human MM cell cultures. As shown in Fig. S1, BH4 treatment also significantly inhibited Bor-induced MM cell apoptosis in both ARP-1 and CAG MM cell models. These results demonstrated that BH4 inhibits Bor-induced MM cell apoptosis in vitro.

To examine the role of BH4 in Bor-induced inhibition of MM tumor growth, BH4 were injected i.p. into MCP-11-bearing mice during Bor treatment. While Bor treatment potently inhibited MPC-11 MM tumor growth as compared with PBS control (Fig. 2b), BH4 treatment largely abolished Bor-induced inhibition of MPC-11 MM tumor growth (Fig. 2b). Moreover, BH4 treatment also inhibited Bor-induced inhibition of MOPC-315 MM tumor growth in Balb/c mouse model (Fig. 2c). Together, these data demonstrated that BH4 increases Bor resistance in MM therapy.

### BH4 increases MM cell survival via USP7 and USP46

**Fig. 3:**
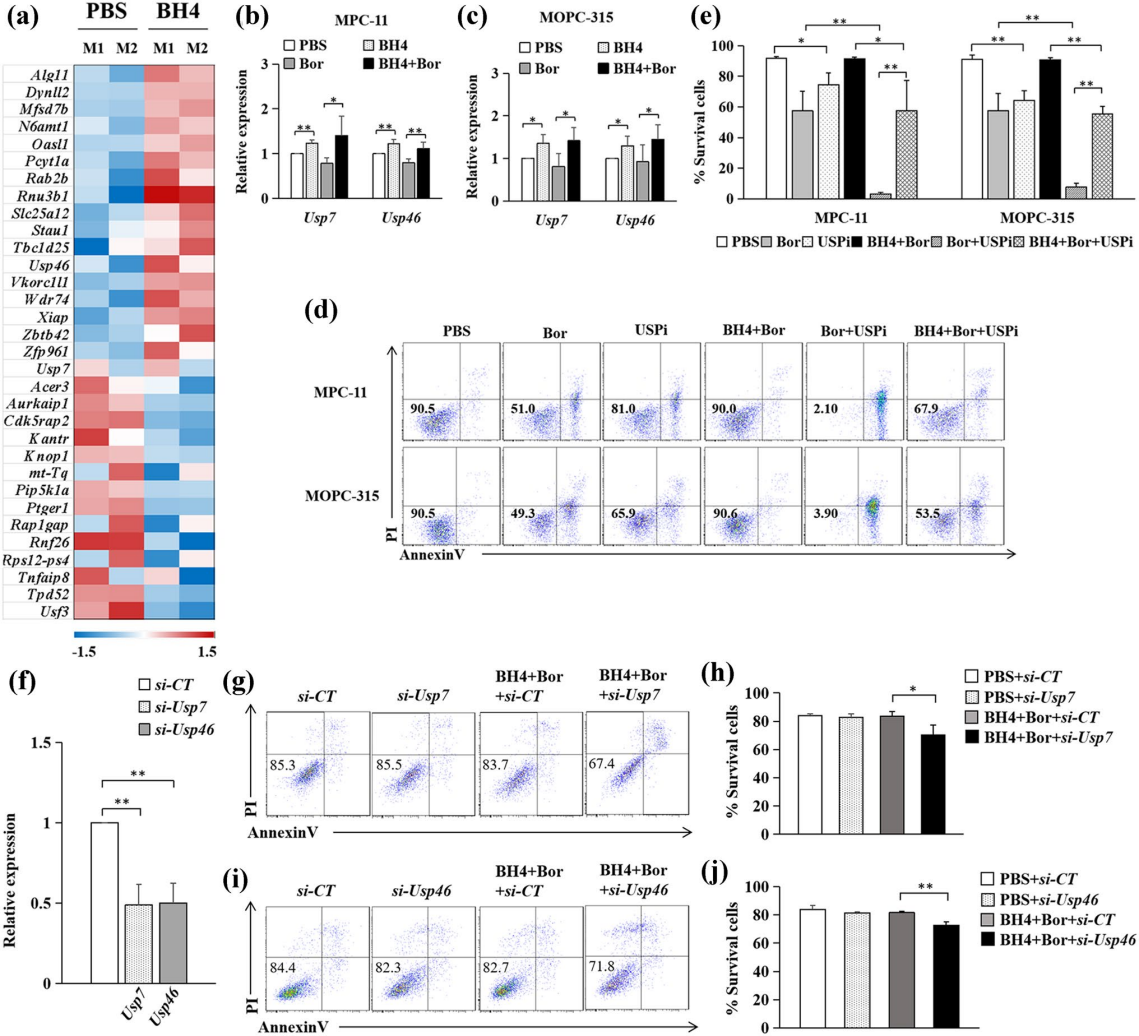
BH4 increases MM cell survival via USP7 and USP46. **a** MPC-11(M1) and MOPC-315(M2) were cultured with or without (PBS) the addition of BH4 for 24 h. Cells were analyzed by RNA-seq. Pink-blue heatmap shows the log2-fold change of the differentially expressed genes. Pink, higher expression; blue, lower expression. **b**, **c** MPC-11 (**b**) and MOPC-315 (**c**) were cultured in the presence or absence (PBS) of BH4, Bor, or their combinations (BH4 + Bor) for 24 h. qPCR assessed the mRNA levels of *Usp7* and*Usp46*. Expression was normalized to *Gapdh* and set at 1 in PBS-treated cells. Results shown are the mean ± SD of three independent experiments. **d** MPC-11 and MOPC-315 cells were cultured in the presence or absence (PBS) of Bor, P22077(USP inhibitor, USPi), BH4 + Bor, Bor + USPi, or their combinations (BH4 + Bor + USPi). Cell apoptosis were analyzed by flow cytometry. Numbers in the dot plots represent viability of MM cells. Showing are representative data of three independent experiments. **e** Summarized results of three independent experiments obtained as (**d**). **f** MPC-11 were treated by *Usp7* (*si-Usp7*), *Usp46* (*si-Usp46*), or control siRNA (*si-CT*) for 24 h. qPCR assessed mRNA levels of *Usp7* and *Usp46*. **g**, **h** MPC-11 treated with *si-Usp7* or *si-CT* were cultured in the presence of BH4 + Bor combinations for 24 h. Cell apoptosis were analyzed by flow cytometry (**g**). Numbers in the dot plots represent viability of MM cells. Showing are representative data of three independent experiments (**h**). **i**, **j** MPC-11 treated with *si-Usp46* or *si-CT* were cultured in the presence of BH4 + Bor combinations for 24 h. Cell apoptosis were analyzed by flow cytometry (**i**). Numbers in the dot plots represent viability of MM cells. Showing are representative data of three independent experiments (**j**). Data are presented as mean ± SD. *P < 0.05; **P < 0.01

We next exploit the molecular mechanism underlying BH4-induced MM cell survival. We first performed RNA-seq analysis in MPC-11 and MOPC-315 MM cells with or without BH4 treatment. We identified 32 differentially expressed genes in MM cells versus BH4-treated MM cells (Fig. 3a).

The protein–deubiquitylation promotes tumor progression and drug resistance through p53 degradation and NF-κB activation [8, 9, 20]. Ubiquitin-specific protease 7 (USP7) inhibits the degradation of MDM2, which upregulates the ubiquitination of p53 and tumor progression [20]. Interestingly, RNA-seq analysis revealed that the addition of BH4 increased the expression of *Usp7* and *Usp46* in MPC-11 and MOPC-315 MM cells (Fig. 3a), suggesting that USP7 and USP46 may mediate BH4-induced MM cell survival. We confirmed the upregulation of *Usp7* and *Usp46* in BH4-treated MPC-11 (Fig. 3b) and MOPC-315 (Fig. 3c) MM cells by qPCR. Moreover, the addition of BH4 also increased the expression of *Usp7* and *Usp46* in Bor-treated MPC-11 (Fig. 3b) and MOPC-315 (Fig. 3c) MM cells. These results indicated that BH4 stimulates the expression of USP7 and USP46 in MM cells.

To address the role of USP7 and USP46 in BH4-treated MM cell survival, an inhibitor of USP family members (especially USP7), P22077 (USPi) was used. As shown in Fig. 3d, the addition of USPi increased MPC-11 MM cell apoptosis as compared with PBS control (Fig. 3d, e), and the addition of USPi further increased the apoptosis of Bor-treated MPC-11 MM cells (Fig. 3d, e). Furthermore, the addition of USPi partially abolished BH4-induced MM cell survival in Bor-treated MM cells (Fig. 3d, e). Furthermore, the addition of USPi also partially abolished the survival of cells treated with BH4 plus Bor compared to Bor alone (Fig. 3d, e). By using MOPC-315 MM cell model, we observed similar results of USPi on BH4-induced MM cell survival (Fig. 3d, e). In addition, by using small interfering RNAs (siRNAs) to specifically silence *Usp7* or *Usp46* in MPC-11 cells (Fig. 3f), we found that knockdown of either *Usp7* or *Usp46* reduced BH4-mediated Bor resistance in MPC-11 cells (Fig. 3g–j). Together, these results indicated that BH4 mediates MM cell survival through USP7 and USP46.

### BH4 increases p53 degradation and NF-κB activation via USP7 and USP46

**Fig. 4:**
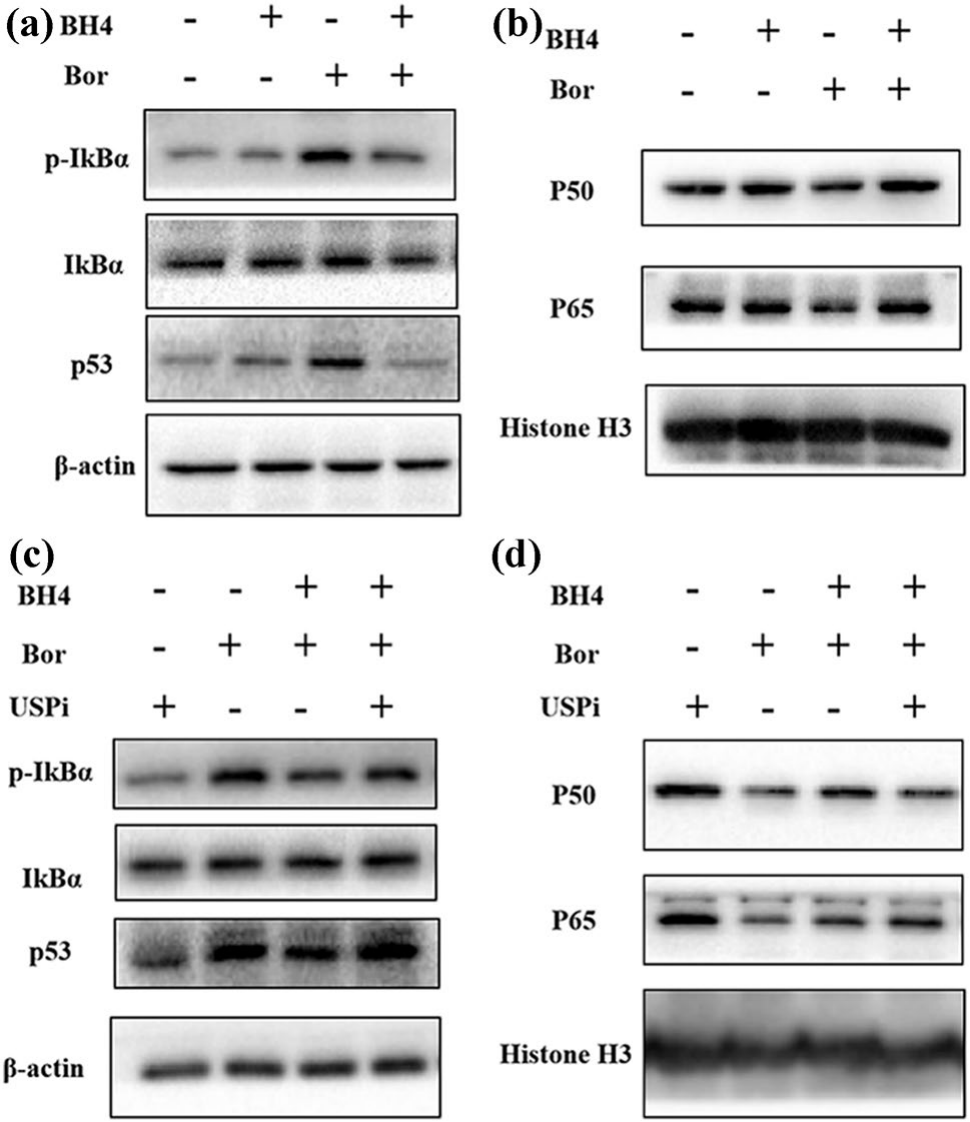
BH4 increases P53 degradation and NF-κB activation via USP7 and USP46. **a** MPC-11 were cultured with or without the additions of BH4, Bor, or their combinations (BH4 + Bor) for 2 h. Western-blots examined the protein levels of pIkBα, IkBα, and p53. β-actin was used as a loading control. The samples derive from the same experiment and that gels/blots were processed in parallel. **b** MPC-11 were cultured with or without the additions of BH4, Bor, or their combinations (BH4 + Bor) for 5 h. The nuclear protein levels of P50 and P65 were examined by western-blots. Histone H3 was used as a loading control. **c** MPC-11 were cultured with USPi, Bor, BH4 + Bor, or BH4 + Bor + USPi for 2 h. Western-blots examined the protein levels of pIkBα, IkBα, and p53. The samples derive from the same experiment and that gels/blots were processed in parallel. **d** MPC-11 were cultured with USPi, Bor, BH4 + Bor, or BH4 + Bor + USPi for 5 h. The nuclear protein levels of P50 and P65 were examined by western-blots. Data are representative (**a**–**d**) of three independent experiments

We next examined the effects of BH4 treatment on p53 and NF-κB signaling. We first explored the role of BH4 in the degradation of p53 in MPC-11 MM cells. As shown in Fig. 4a, BH4 treatment exhibited minor effects on the protein level of p53 in MM cells as compared to PBS control. However, as expected, Bor treatment increased the protein level of p53 in MM cells as compared to PBS control (Fig. 4a), and the addition of BH4 almost completely abolished the increase of p53 in Bor-treated MM cells (Fig. 4a). We then examined the role of BH4 treatment in NF-κB signaling in MM cells. Similarly, BH4 treatment exhibited minor effects on the protein levels of p-IκBα and IκBα (Fig. 4a), but slightly increased the nuclear translocation of p50 and p65 in MM cells as compared to PBS control (Fig. 4b). And, Bor treatment increased the protein levels of p-IκBα and IκBα (Fig. 4a), and decreased the nuclear translocation of p50 and p65 in MM cells as compared with PBS control (Fig. 4b). However, BH4 treatment markedly decreased the protein levels of p-IkBα and IkB-α (Fig. 4a) and increased the nuclear translocation of p50 and p65 in Bor-treated MM cells (Fig. 4b). These data indicated that BH4 promotes p53 degradation and NF-κB activation in MM cells.

To determine the role of USP7 and USP46 in BH4-mediated p53 and NF-κB signaling, an USP inhibitor (P22077, USPi) was used. We found that USPi partially abolished BH4-induced decrease of p53, p-IκBα, and IκBα in Bor-treated MM cells (Fig. 4c). Furthermore, USPi partially abolished BH4-induced increase of p50 and p65 nuclear translocation in Bor-treated MM cells (Fig. 4d), indicating that USPi inhibited BH4-induced p53 degradation and NF-κB activation in MM cells. Collectively, these data indicated that BH4 mediates p53 degradation and NF-κB activation through USP7 and USP46.

### The inhibition of USPs promotes therapeutic effects of Bor in MM

**Fig. 5:**
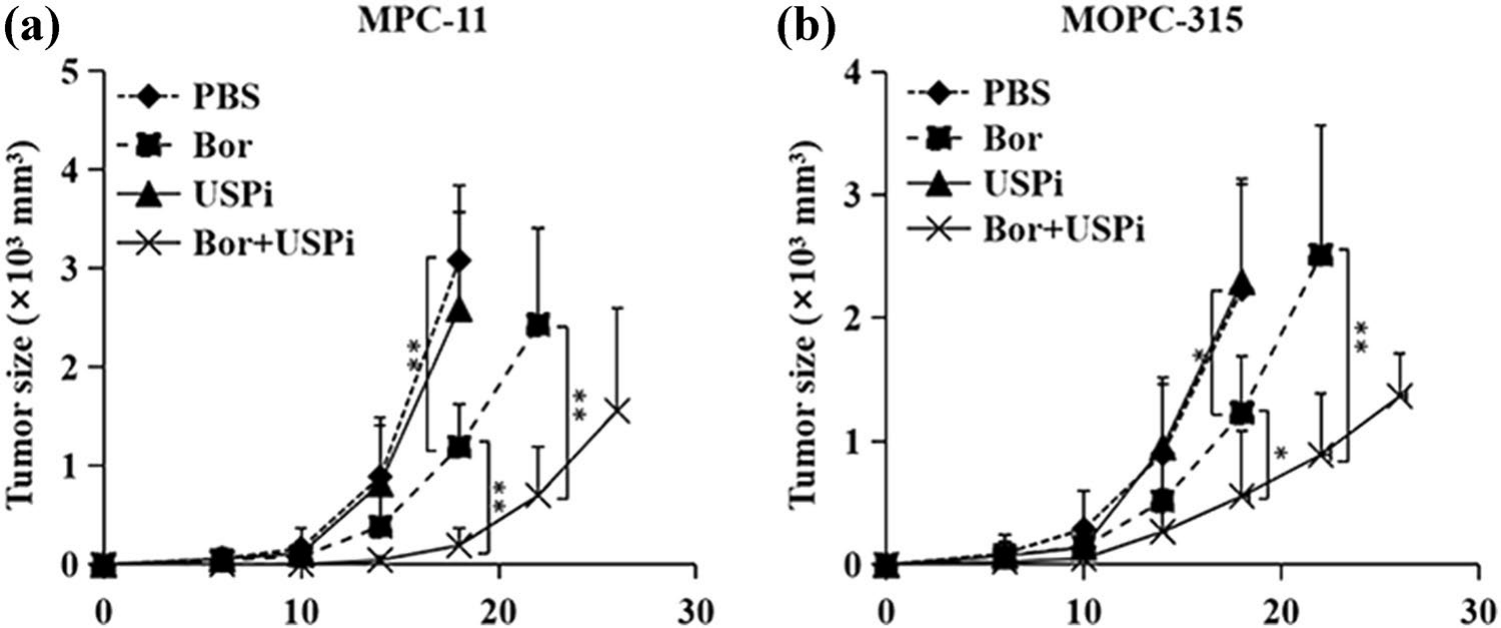
The inhibition of USPs promotes therapeutic effects of Bor in MM. Balb/c mice were injected s.c. with MPC-11 (**a**) or MOPC-315 (**b**) cells (1 × 10^6^ per mouse). From day 6 after tumor challenge, mice were injected i.p. with Bor, USPi, or their combinations (Bor + USPi) every 2 days. Mice received PBS served as controls. Shown are the tumor growth curves. The experiments were performed twice with a total of 10 mice per group (*n* = 10). Data are presented as mean ± SD. *P < 0.05; **P < 0.01

To examine the role of USPs in MM therapy, USP inhibitor was used during Bor-mediated MM treatment. MPC-11 cells were injected s.c. into Balb/c mice and mice were treated with USP inhibitor and Bor. While USPi treatment exhibited minor effects on MM tumor growth as compared to PBS control, the addition of USPi increased Bor-mediated inhibition of MM tumor growth (Fig. 5a). Furthermore, the combination of USPi and Bor also induced more potent antitumor response than Bor alone in MOPC-315 MM Balb/c mouse model (Fig. 5b). Thus, our results demonstrated that the inhibition of USPs increased Bor antitumor effects in MM.

## Discussion

MM still remains an incurable disease due to widespread drug resistance and high frequency of relapse [4]. Therefore, identifying factors associated with MM drug resistance and progression may have important clinical significance. In this study, we found that BH4 treatment promoted MM cell proliferation and tumor growth in vivo. In addition, BH4 treatment largely abrogated Bor-induced MM cell apoptosis in vitro. Furthermore, the addition of BH4 decreased Bor-mediated inhibition of MM tumor growth in vivo. Thus, our data indicated a potential role of BH4 in MM drug resistance and tumor progression.

Ubiquitin-specific proteases (USPs) include USP6, USP7, USP46, USP47 et al., which are involved in protein deubiquitylation and inhibit proteasome-mediated protein degradation [6]. In this study, we found that BH4 treatment increased the expressions of *Usp7* and *Usp46* in MM cells. In addition, the inhibition of USPs decreased BH4-induced MM Bor resistance and increased Bor-induced MM cell apoptosis in vitro. Furthermore, USP inhibition increased the therapeutic efficacy of Bor in MM-bearing mice. Interestingly, USP7 also promotes tumorigenesis in breast cancer and cervical cancer [21, 22]. These observations indicate that BH4 mediates MM drug resistance and cell survival through USP7 and USP46.

In this study, we found that BH4 promoted the degradation of p53 and IkBα in Bor-treated MM cells and the inhibition of USPs reduced BH4-mediated degradation of p53 and IkBα in Bor-treated MM cells. Interestingly, USP7 is also shown to stabilize MDM2 and NEK2 kinase which contribute to the degradation of p53 and the activation of NF-κB signaling pathway in other cancers [20, 23]. These observations suggest that BH4 promotes MM Bor resistance and tumor progression via the degradation of p53 and the activation of NF-κB signaling pathway.

In this study, we found that the inhibition of USPs only partially abrogated BH4-induced MM cell survival in Bor-treated MM cells. BH4 up-regulates proteasome activity in a NO-dependent manner [15, 24]. These investigations suggest that the NO-mediated up-regulation of proteasome activity may also contribute to BH4-induced MM Bor resistance and tumor progression. Further studies will be necessary to investigate the role of proteasome activity in BH4-induced MM Bor resistance and tumor progression.

## Conclusion

In summary, our study suggests that BH4 treatment potently promotes MM progression in vivo*.* BH4 treatment inhibits Bor-induced antitumor effects. BH4 increases p53 degradation and activates NF-kB signaling pathways in a USP-dependent manner, and the inhibition of USPs increases Bor-mediated antitumor effects in mouse models. Our results identified BH4 as a powerful inducer of MM progression and Bor resistance and clarified the underlying mechanisms.

## Supplementary Information

Below is the link to the electronic supplementary material.

Fig. S1 BH4 promotes Bor resistance in human MM cell lines ARP-1 and CAG cells were cultured in the presence of BH4, Bor or their combinations (BH4+Bor) for 24 hours. Cells treated with PBS served as controls. Cell apoptosis was analyzed by Flowcytometry. Numbers in the dot plots represent viability of MM cells. Showing are representative data of three independent experiments. Right, summarized results of three independent experiments obtained as left. Data are presented as mean ± SD. * P<0.05; ** P<0.01. Supplementary file1 (TIF 1211 KB)

## Data Availability

The datasets used and/or analyzed during the current study are available from the corresponding author on reasonable request.

## Abbreviations

BH4: Tetrahydrobiopterin
MM: Multiple Myeloma
BTZ: Bortezomib
NF-κB: Nuclear factor kappa-B
FBS: Fetal bovine serum
qPCR: Real time quantitative polymerase chain reaction
USP: Ubiquitin-specific proteases
FI: Fluorescence intensity
MFI: Mean fluorescence intensity
