# Supplementary figures and images for: Tetrahydrobiopterin induces proteasome inhibitor resistance and tumor progression in multiple myeloma
Source: Med Oncol. 2022 Feb 12;39(5):55. doi: 10.1007/s12032-021-01632-5 (PMC8840911; doi:10.1007/s12032-021-01632-5)

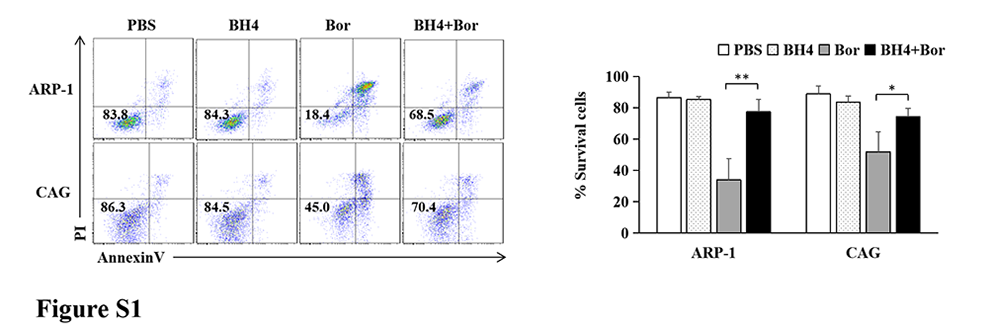

Supplement: Supplementary file 1 — Fig. S1 BH4 promotes Bor resistance in human MM cell lines ARP-1 and CAG cells were cultured in the presence of BH4, Bor or their combinations (BH4+Bor) for 24 hours. Cells treated with PBS served as controls. Cell apoptosis was analyzed by Flowcytometry. Numbers in the dot plots represent viability of MM cells. Showing are representative data of three independent experiments. Right, summarized results of three independent experiments obtained as left. Data are presented as mean ± SD. * P<0.05; ** P<0.01. Supplementary file1 (TIF 1211 KB) [file 12032_2021_1632_MOESM1_ESM.tif]
